# Statement of Retraction: Long non-coding RNA MEG3 suppresses the development of bladder urothelial carcinoma by regulating miR-96 and TPM1

**DOI:** 10.1080/15384047.2026.2740300

**Published:** 2026-09-29

We, the Editors and Publisher of *Cancer Biology & Therapy*, have retracted the following article:

Liu, G., Zhao, X., Zhou, J., Cheng, X., Ye, Z., & Ji, Z. (2018). Long non-coding RNA MEG3 suppresses the development of bladder urothelial carcinoma by regulating miR-96 and TPM1. *Cancer Biology & Therapy*, *19*(11), 1039–1056. https://doi.org/10.1080/15384047.2018.1480279

Following publication, concerns were raised regarding the image integrity of Flow Cytometry Figures 2C, 6C, 10C, Western Blot Figures 9F, Tumour Figures 11A and the qRT-PCR methodology and results described in the article. When approached for an explanation, the authors did not respond to our queries.

As verifying the validity of published work is core to the integrity of the scholarly record, we are therefore retracting the article. The corresponding author has been informed.

We have been informed in our decision-making by our editorial policies and the COPE guidelines.

The retracted article will remain online to maintain the scholarly record, but it will be digitally watermarked on each page as ‘Retracted’.

